# MMP-3 Deficiency Alleviates Endotoxin-Induced Acute Inflammation in the Posterior Eye Segment

**DOI:** 10.3390/ijms17111825

**Published:** 2016-11-01

**Authors:** Inge Van Hove, Evy Lefevere, Lies De Groef, Jurgen Sergeys, Manuel Salinas-Navarro, Claude Libert, Roosmarijn Vandenbroucke, Lieve Moons

**Affiliations:** 1 Neural Circuit Development and Regeneration Research Group, Department of Biology, Katholieke Universiteit Leuven (KU Leuven), B-3000 Leuven, Belgium; inge.vanhove@kuleuven.be (I.V.H.); evy.lefevere@kuleuven.be (E.L.); lies.degroef@kuleuven.be (L.D.G.); jurgen.sergeys@kuleuven.be (J.S.); manuel.salinasnavarro@kuleuven.be (M.S.-N.); 2 Laboratory of Experimental Ophthalmology, Department of Neurosciences, KU Leuven, B-3000 Leuven, Belgium; 3 Inflammation Research Center, VIB, B-9052 Ghent, Belgium; claude.libert@irc.vib-ugent.be (C.L.); roosmarijn.vandenbroucke@irc.vib-ugent.be (R.V.); 4 Department of Biomedical Molecular Biology, Ghent University, B-9052 Ghent, Belgium

**Keywords:** LPS, MMP-3, inflammation, retina, blood–retinal barrier, retinal pigment epithelium, leukostasis

## Abstract

Matrix metalloproteinase-3 (MMP-3) is known to mediate neuroinflammatory processes by activating microglia, disrupting blood–central nervous system barriers and supporting neutrophil influx into the brain. In addition, the posterior part of the eye, more specifically the retina, the retinal pigment epithelium (RPE) and the blood–retinal barrier, is affected upon neuroinflammation, but a role for MMP-3 during ocular inflammation remains elusive. We investigated whether MMP-3 contributes to acute inflammation in the eye using the endotoxin-induced uveitis (EIU) model. Systemic administration of lipopolysaccharide induced an increase in MMP-3 mRNA and protein expression level in the posterior part of the eye. MMP-3 deficiency or knockdown suppressed retinal leukocyte adhesion and leukocyte infiltration into the vitreous cavity in mice subjected to EIU. Moreover, retinal and RPE mRNA levels of intercellular adhesion molecule 1 (*Icam1*), interleukin 6 (*Il6*), cytokine-inducible nitrogen oxide synthase (*Nos2*) and tumor necrosis factor α (*Tnfα*), which are key molecules involved in EIU, were clearly reduced in MMP-3 deficient mice. In addition, loss of MMP-3 repressed the upregulation of the chemokines monocyte chemoattractant protein (MCP)-1 and (C-X-C motif) ligand 1 (CXCL1). These findings suggest a contribution of MMP-3 during EIU, and its potential use as a therapeutic drug target in reducing ocular inflammation.

## 1. Introduction

The eye has evolved into a specialized, tightly regulated immune privileged organ with structural and functional mechanisms that need to protect the integrity of the neural retina and maintain retinal homeostasis. The blood–retinal barrier (BRB) performs a crucial function to maintain this ocular immune privilege. Both inner and outer components of the BRB, formed by, respectively, endothelial and retinal pigment epithelial (RPE) cells, physically separate the retina from the immune system and prevent leukocyte infiltration from the blood vessels into the retina. Next to the BRB, eye-specific mechanisms and the specialized, unique intraocular microenvironment also contribute to reducing systemic and local immune responses [1]. However, acute inflammatory processes deteriorate into severe damage of BRB structure and functionality and result in distortion of the retinal microenvironment. Indeed, inflammation-induced pathogenic mechanisms contribute to vision-threatening diseases such as diabetic retinopathy, retinitis pigmentosa, glaucoma, age-related macular degeneration (AMD) and uveitis [1,2]. Therefore, investigating the mechanisms by which the BRB/retina responds to inflammatory stimuli is an important research question, and of crucial importance for the development of novel therapies for multiple retinal inflammatory diseases.

Acute systemic ocular inflammation can be induced in rodents using the endotoxin-induced uveitis (EIU) model, i.e., by intraperitoneal injection of the endotoxin lipopolysaccharide (LPS), the major component of the outer membrane of gram-negative bacteria. This model mainly mimics acute inflammatory responses, driven by innate immune mechanisms [1], and is therefore invaluable in unraveling the ocular inflammatory mediators. Besides its power to investigate acute vascular inflammation at the uveal tract [3], EIU is also a compelling tool to evaluate inflammatory responses at the posterior part of the eye. It is characterized by an increment in adhesion of leukocytes to the retinal vasculature and infiltration of leukocytes into the retina/vitreous cavity [4,5]. Increased oxidative stress, inflammatory cytokines (e.g., interleukin (IL)-6 and tumor necrosis factor (TNF)-α) and chemokines (e.g., monocyte chemoattractant protein (MCP)-1 and C-X-C motif ligand (CXCL) 1/2), and adhesion molecules (e.g., soluble vascular cell adhesion molecule (sVCAM) and intracellular adhesion molecules (ICAMs)) are key inflammatory mediators that contribute to the pathology of rodent EIU [5,6,7,8,9,10].

Several reports describe that matrix metalloproteinases (MMPs), i.e., Zn^2+^-dependent endopeptidases that cleave both matrix and non-matrix proteins, are able to degrade basal lamina components and tight junction proteins at the blood–brain barrier (BBB), blood–cerebrospinal fluid barrier (BCSFB) and blood–spinal cord barrier (BSCB), thereby contributing to blood–central nervous system (CNS) barrier disruption and neuroinflammation [11,12,13,14,15,16,17,18,19]. Of note, it is known that the endothelial cells of the BBB/BSCB and the choroid plexus epithelial cells (CPE) that form the BCSFB show similarities with, respectively, the endothelial cells of the inner BRB and the RPE cells of the outer BRB [20]. In the brain, MMP expression is known to be upregulated by cytokines but also by the bacterial endotoxin LPS. In addition, increased MMP-3 levels have been reported during neuroinflammatory processes [11,14,21,22,23,24,25,26]. Indeed, the MMP-3 gene (*Mmp3*) promoter contains binding sites for activator protein 1 (AP-1) and nuclear factor kappa-light-chain-enhancer of activated B cells (NF-κB), which can both be activated by various inflammatory cytokines [27,28,29,30]. Upregulated MMP-3 expression/activity is then suggested to result in an impairment of the blood–CNS barriers due to its effects on degradation of tight junction proteins and/or the basal lamina, but also by activating microglia and stimulating expression and activation of cytokines, thereby supporting neutrophil influx [11,16,25,31,32,33]. Notably, MMP-3 is shown to be expressed by all cell types in the brain as well as by blood mononuclear cells [31]. Moreover, cultured human retinal microvascular endothelial cells and RPE cells exposed to TNF-α secrete more MMP-3 protein [34,35]. In addition, significantly increased MMP-3 mRNA levels are detected in RPE/choroid samples of age-related macular degeneration (AMD) patients, indicating its importance in ocular inflammatory processes [36].

The increasing knowledge of MMP-3 as a crucial mediator of the blood–CNS barrier integrity during neuroinflammatory conditions suggests its potential contribution to systemic inflammation-induced alterations at the retina/BRB. In the present study, we investigated the involvement of MMP-3 in endotoxin-induced acute inflammation in the posterior part of the mouse eye.

## 2. Results

### 2.1. Endotoxemia Induces Upregulation of Matrix Metalloproteinase-3 (MMP-3) mRNA and Protein Levels in the Posterior Part of the Eye

**Figure 1:**
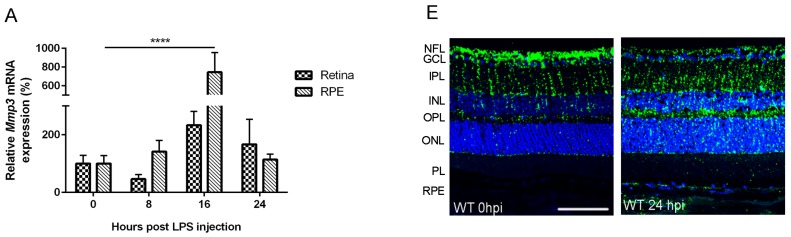

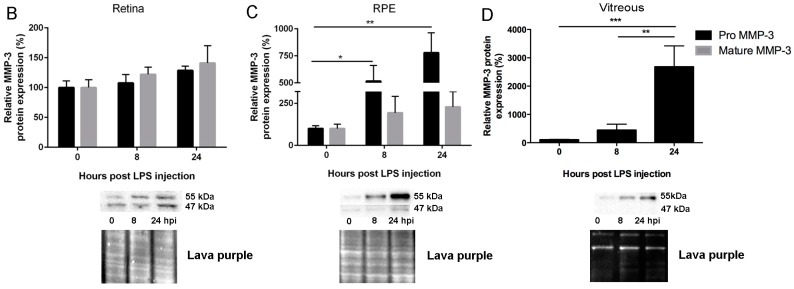
Matrix metalloproteinase-3 (MMP-3) mRNA and protein spatiotemporal expression pattern in the retina, retinal pigment epithelium (RPE) and vitreous of mice subjected to endotoxin-induced uveitis (EIU). (**A**) Real time-PCR analysis on retina and RPE samples from wild-type (WT) mice subjected to EIU revealed an apparent upregulation of *Mmp3* levels in the RPE, reaching a peak around 16 hpi (hour post LPS injection), and returning to baseline levels at 24 hpi. Retinal samples demonstrated a similar upregulation in *Mmp3* mRNA levels, although to a much lesser extent. RT-PCR data were normalized against the reference genes and were expressed as a percentage relative to 0 hpi; (**B**,**C**) Western blot data confirmed the PCR findings and showed a slight increase in pro- and mature MMP-3 levels in the retina, but a more pronounced upregulation of, in particular, pro MMP-3 protein in the RPE; (**D**) Western blot data revealed a significant increase of pro MMP-3 levels after 8 and 24 hpi in the vitreous cavity. Lava purple staining served as a loading control; (**E**) Representative images show localization of MMP-3 immunoreactivity (green) in the Müller glia in the retina of healthy WT mice. The blue staining represents DAPI nuclear counterstain. EIU increased expression of MMP-3 in these glial fibers, but also in the RPE. Scale bar: 100 µm. Western blot data were normalized against Lava purple total protein stain and expressed as a percentage relative to 0 hpi. All data are shown as mean ± SEM, *n* ≥ 5, * *p* ≤ 0.05, ** *p* ≤ 0.01, *** *p* ≤ 0.001, **** *p* ≤ 0.0001. NFL, nerve fiber layer; GCL, ganglion cell layer; IPL, inner plexiform layer; INL, inner nuclear layer; OPL, outer plexiform layer; ONL, outer nuclear layer; PL, photoreceptor layer; RPE, retinal pigment epithelium.

To evaluate whether MMP-3 is important during EIU in the posterior segment of the eye, the MMP-3 mRNA and protein levels were determined at different time points post LPS injection. Real time-PCR data revealed rather low levels of *Mmp3* in the RPE and the retina at baseline (0 hour post LPS injection (hpi)) (Figure 1A). However, after induction of endotoxemia, an increment in *Mmp3* mRNA levels was observed, especially in the RPE, with a peak at 16 hpi and a decline thereafter. Indeed, *Mmp3* exhibited a 2.3- (*p* = not significant (NS)) and a 7.4-fold (*p* ≤ 0.0001) increase in mRNA expression in the retina and RPE, respectively, at 16 hpi following LPS treatment. Consistent with these findings, pro MMP-3 (55 kDa) protein levels were slightly augmented in the retina (Figure 1B), but significantly elevated in the RPE at 8 and 24 hpi by 5.2- and 7.8-fold, respectively (Figure 1C). Mature MMP-3 levels showed a trend towards an increased expression in both the retina and RPE after LPS injection (Figure 1B,C). Interestingly, pro MMP-3 significantly increased in the vitreous cavity at 8 and 24 hpi by 4.4- and 26-fold respectively (Figure 1D), suggesting a translocation of MMP-3 to the vitreous after induction of endotoxemia. To further localize MMP-3 in the posterior eye segment, immunohistochemistry was performed. MMP-3 protein expression at baseline was observed in the endfeet and radial fibers of the Müller glia (Figure 1E). In the retinas of mice with EIU, the MMP-3 immunopositive signal was most dramatically enhanced in the outer retina, more specifically in the radial fibers of the Müller glia, and in the RPE/choroid complex. Together, these data point to a boost of MMP-3 expression after endotoxemia in the posterior segment of the eye, predominantly in the RPE, and indicate a potential role of MMP-3 in LPS-induced inflammatory processes.

### 2.2. MMP-3 Deficient Mice Do Not Show an Aberrant Retinal Morphology or Visual Acuity

**Figure 2:**
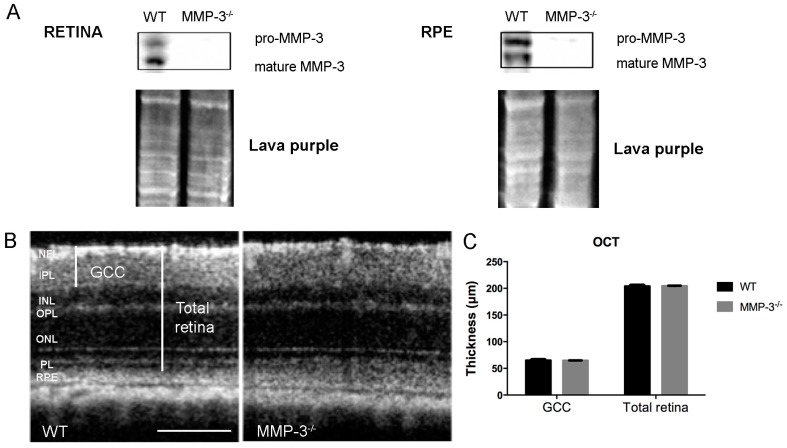

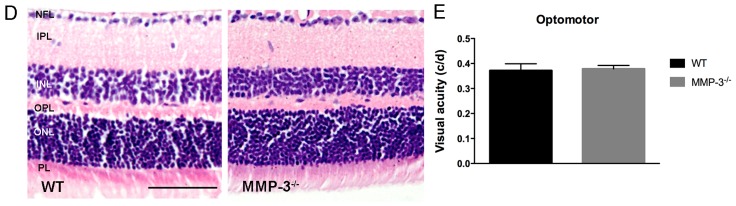
Retinal morphology and visual acuity of healthy WT and MMP-3^−/−^ mice. (**A**) Absence of pro- and mature MMP-3 in the retina and RPE of MMP-3^−/−^ mice was confirmed via Western blotting. Lava purple staining served as a loading control; (**B**) Representative spectral domain optical coherence tomography (SD-OCT) pictures showed no difference in general morphology of the retina in WT and MMP-3^−/−^ mice. Scale bar: 100 µm; (**C**) Analysis of SD-OCT images revealed a similar thickness of the ganglion cell complex (GCC), which includes the nerve fiber layer (NFL), ganglion cell layer (GCL) and inner plexiform layer (IPL), and of the total retina in both genotypes; (**D**) Representative images of hematoxylin and eosin (H&E) stained retinal sections demonstrated no difference in the thickness of the retinal layers in WT and MMP-3^−/−^ eyes. Scale bar: 50 µm; (**E**) Visual acuity, assessed in a virtual optomotor device, was found to be similar in WT and MMP-3^−/−^ mice. Data are shown as mean ± SEM, *n* ≥ 8. NFL, nerve fiber layer; IPL, inner plexiform layer; INL, inner nuclear layer; OPL, outer plexiform layer; ONL, outer nuclear layer; PL, photoreceptor layer; RPE, retinal pigment epithelium.

Absence of MMP-3 in MMP-3 deficient (MMP-3^−/−^) retina and RPE samples was confirmed via Western blotting (Figure 2A). Next, to investigate whether MMP-3 deficiency affects basal retinal development and morphology, spectral domain optical coherence tomography (SD-OCT) was performed on wild-type (WT) and MMP-3^−/−^ mouse eyes. OCT scans demonstrated no obvious morphological differences in the retina of adult MMP-3^−/−^ compared to WT animals (Figure 2B). In addition, a more detailed morphometric analysis revealed no discrepancies in the thickness of the ganglion cell complex (GCC) and the total retinal thickness, indicating that MMP-3 deficiency does not affect retinal architecture (Figure 2C). These findings were confirmed via hematoxylin and eosin (H&E) staining on retinal sections (Figure 2D). Indeed, morphometric analysis on H&E stained sections showed no difference in the thickness of the retinal layers (mean thickness from NFL till ONL: 134.1 ± 4.7 µm in MMP-3^−/−^ mice vs. 147.6 ± 6.4 µm in WT mice, *n* = 3). Furthermore, retinal functionality was evaluated via the optokinetic tracking response. Visual acuity, measured as the highest spatial frequency (cycles/degree) eliciting an optokinetic response, showed no significant difference between the genotypes (Figure 2E). To conclude, assessment of the retinal morphology and the optokinetic tracking response revealed that vision and normal eye function are preserved in adult MMP-3^−/−^ mice.

### 2.3. MMP-3 Deficiency and Knockdown Attenuates the Hypothermic Response after Endotoxemia

**Figure 3:**
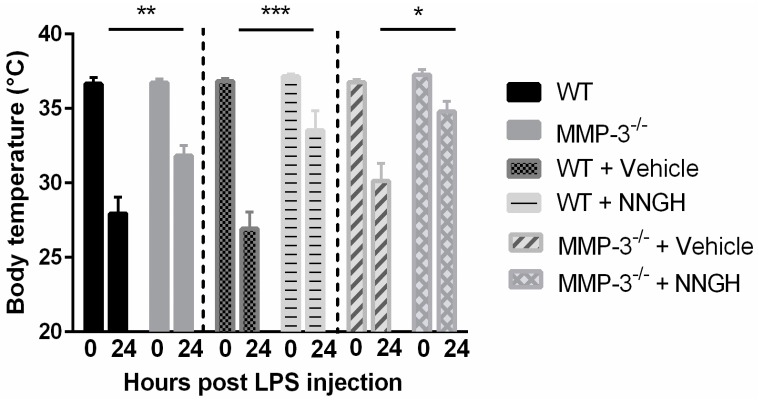
Influence of MMP-3 deficiency on body temperature during EIU, observed during independent experiments (indicated by dotted lines). At 24 h post LPS injection, body temperature clearly dropped in naive- and vehicle- (DMSO 5%) treated WT mice. MMP-3 deficiency resulted in a reduced sensitivity for LPS as shown by the significantly higher body temperature in MMP-3^−/−^ and *N*-Isobutyl-*N*-(4-methoxyphenylsulfonyl)-glycyl hydroxamic acid (NNGH)-injected mice, as compared to WT and vehicle-treated animals, respectively. Treatment of MMP-3^−/−^ mice with NNGH resulted in a more pronounced attenuation in the hypothermic response as compared to vehicle-treated MMP-3^−/−^ mice. Data are shown as mean ± SEM, *n* ≥ 5, * *p* ≤ 0.05, ** *p* ≤ 0.01, *** *p* ≤ 0.001.

To investigate the role of MMP-3 in acute inflammation in the retina and RPE, WT and MMP-3^−/−^ animals were subjected to an experimental model of bacterial endotoxin-induced systemic inflammation, which is known to induce a hypothermic response in mice, also at sub-lethal doses [37]. A profound drop in body temperature is indeed visible in both WT and MMP-3^−/−^ mice at 24 hpi, however, body temperature is significantly higher in MMP-3^−/−^ as compared to WT animals (31.8 ± 0.7 °C in MMP-3^−/−^ vs. 27.9 ± 1.1 °C in WT mice, *p* ≤ 0.01) (Figure 3), suggesting that MMP-3^−/−^ mice are less sensitive to LPS-induced hypothermia. This protective effect of MMP-3 deficiency was confirmed after administration of *N*-Isobutyl-*N*-(4-methoxyphenylsulfonyl)-glycyl hydroxamic acid (NNGH), a commonly used semi-selective MMP-3 inhibitor [32,38,39,40], in WT animals. Based on the observed MMP-3 expression profile, NNGH (1.6 µg/µL) was injected intraperitoneally 15 min before, and at 6 and 14 hpi. As compared to vehicle (5% DMSO), NNGH-injected mice demonstrated an attenuated hypothermic response, which was similar, or even more pronounced, as compared to the findings in MMP-3^−/−^ mice (33.5 ± 1.3 °C in NNGH-treated vs. 26.9 ± 1.1 °C in vehicle-treated WT mice, *p* ≤ 0.001) (Figure 3). As such, MMP-3 deficiency/reduction clearly attenuates LPS-induced hypothermia, supporting a devastating role of MMP-3 in endotoxemia and its contributing inflammatory processes in the CNS. However, when MMP-3^−/−^ mice were treated with NNGH, body temperature remained higher as compared to vehicle-treated MMP-3^−/−^ animals at 24 hpi (34.8 ± 0.7 °C in MMP-3^−/−^ NNGH-treated mice vs. 30.1 ± 1.2 °C in MMP-3^−/−^ vehicle-treated mice, *p* ≤ 0.05) (Figure 3). Inhibition with NNGH thus shows an additive effect in MMP-3^−/−^ mice, indicating that also other MMPs beside MMP-3 attenuate the hypothermic response induced by systemic LPS administration.

### 2.4. MMP-3 Deficiency or Knockdown Reduces Retinal Leukocyte Adhesion and Vitreous Infiltrating Leukocytes in the Endotoxin-Induced Uveitis (EIU) Model

**Figure 4:**
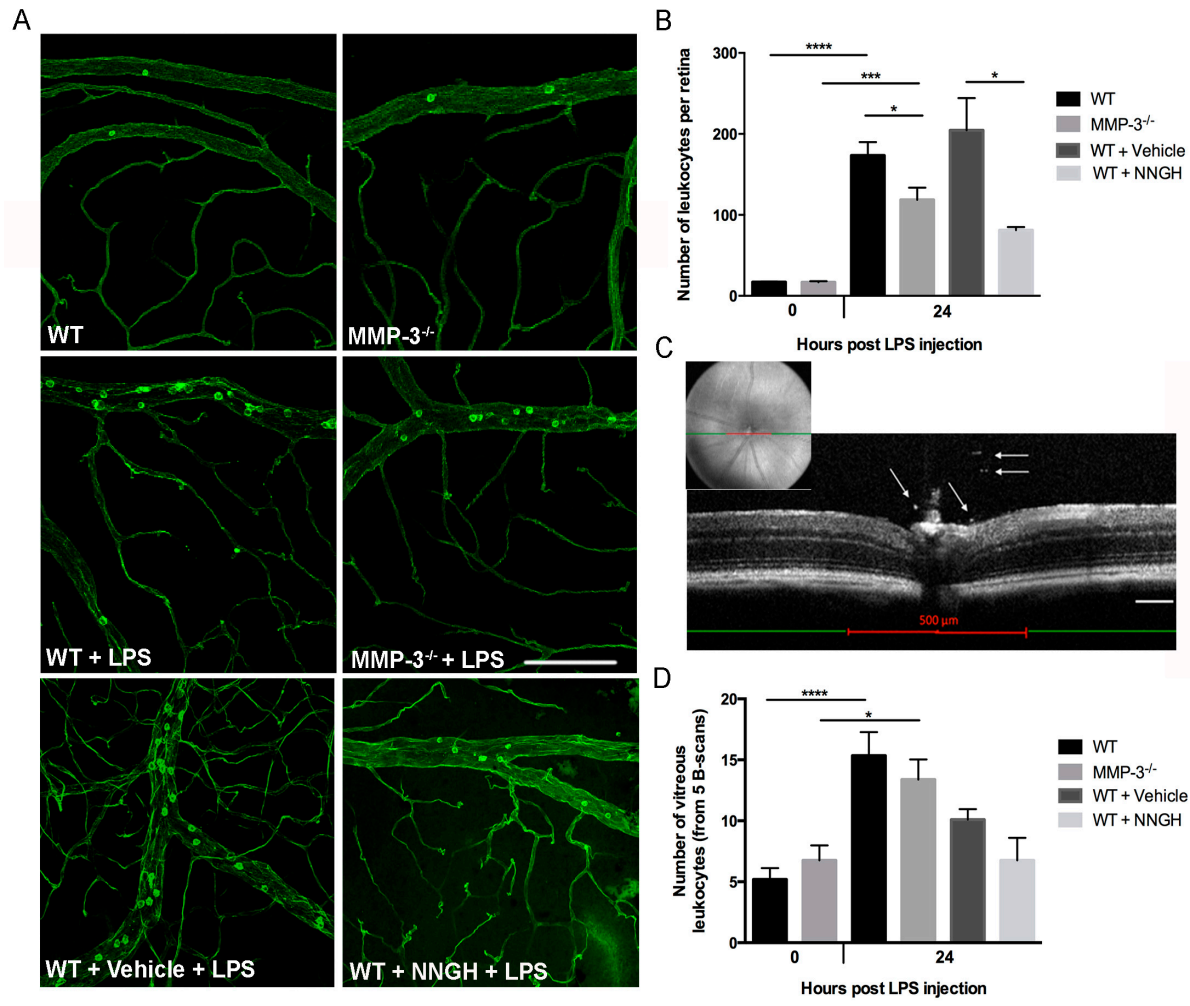
Impact of MMP-3 deficiency on leukocyte adhesion in the retinal vessels and on the amount of infiltrating leukocytes into the vitreous cavity during EIU. (**A**) EIU induces a prominent augmentation in the number of firmly adhering leukocytes at the retinal vessel wall as demonstrated in flatmounted retinas from WT and MMP-3^−/−^ mice at 24 hpi. However, leukocyte adhesion seems to be reduced in MMP-3^−/−^ animals. Similarly, the number of adherent leukocytes seems to be lower in NNGH-treated vs. vehicle-treated WT mice at 24 hpi; Scale bar: 100 µm; (**B**) Quantification of adherent retinal leukocytes in the arterioles and venules showed significantly less adherent leukocytes in EIU-treated MMP-3^−/−^ mice than in WT animals (*n* ≥ 5). In addition, leukostasis was also suppressed after repeated intraperitoneal injections of the MMP-3 inhibitor NNGH, compared to vehicle (5% DMSO)-injected eyes (*n* ≥ 4). * *p* ≤ 0.05, *** *p* ≤ 0.001 and **** *p* ≤ 0.0001; (**C**) OCT measurements at 24 hpi were assessed to evaluate the number of leukocytes infiltrating into the vitreous cavity in healthy and EIU MMP-3^−/−^, WT and NNGH- or vehicle-treated WT mice. Infiltrating leukocytes (as indicated on the single B-scan by arrows) were manually counted on five single B-scan images over a distance of 500 µm (red line), centered on the optic disc using fundus images: one B-scan image at the optic nerve head and four images at 50 µm intervals from the optic nerve head (two anterior and two posterior). Scale bar: 100 µm; (**D**) The number of infiltrating leukocytes in the vitreous significantly increased in both WT and MMP-3^−/−^ mice at 24 hpi. Interestingly, MMP-3 deficiency, either via genetic or pharmacological manipulation, slightly reduced the number of infiltrating leukocytes as compared to (vehicle-treated) WT eyes (*n* ≥ 5). Data are shown as mean ± SEM, * *p* ≤ 0.05, **** *p* ≤ 0.0001.

To study whether the elevated MMP-3 levels in the posterior eye segment mediate acute inflammation in the EIU model, retinal vasculature and adhering leukocytes were labeled by cardiac perfusion with FITC-conjugated Concanavalin A lectin. The number of adherent leukocytes was counted at 0 and 24 hpi in the venules and arterioles on retinal whole mount preparations of both genotypes (Figure 4A). In baseline conditions, no difference in the number of retinal leukocytes was observed in the retina of WT and MMP-3^−/−^ mice (Figure 4A,B). However, as expected, a significant 10-fold increase in the number of adherent leukocytes was noticed in EIU-treated WT mice as compared to healthy WT (Figure 4A,B). Similarly, leukostasis was highly augmented in MMP-3^−/−^ retinas after LPS treatment vs. non-treated MMP-3^−/−^ mice (7-fold increase) (Figure 4A,B), but to a much lower extent (31%, *p* ≤ 0.05) than observed in WT mice after endotoxemia. These findings were confirmed after blocking MMP-3 levels in WT mice via repeated administration of NNGH, which reduced the number of adherent retinal leukocytes by 60% as compared to vehicle-treated mice (Figure 4A,B). As such, blocking MMP-3 results in a reduced leukostasis response in the retinal vasculature during endotoxemia.

To further confirm the inhibitory effect of MMP-3 on acute retinal inflammation, SD-OCT was applied as a non-invasive, in vivo imaging technique to quantify the number of infiltrating leukocytes in the vitreous cavity (adjacent to the optic nerve head) at 24 hpi, in both WT and MMP-3^−/−^ mice (Figure 4C,D) [41,42,43]. The number of leukocytes significantly increased, respectively, by 3- and 2-fold in the vitreous of WT and MMP-3^−/−^ eyes after induction of endotoxemia (Figure 4D). Interestingly, although not significant, MMP-3 deficiency does seem to reduce the number of infiltrating leukocytes in the vitreous by 13% during EIU. These findings were further confirmed using NNGH- and vehicle-treated WT mice, subjected to LPS. Indeed, although not significant due to variability, NNGH treatment decreased the number of infiltrating leukocytes in the vitreous by 33% during EIU (Figure 4D).

### 2.5. Inflammation-Associated Molecules Are Declined in the Retina and Retinal Pigment Epithelial (RPE) of MMP-3 Deficient Mice Subjected to EIU

**Figure 5:**
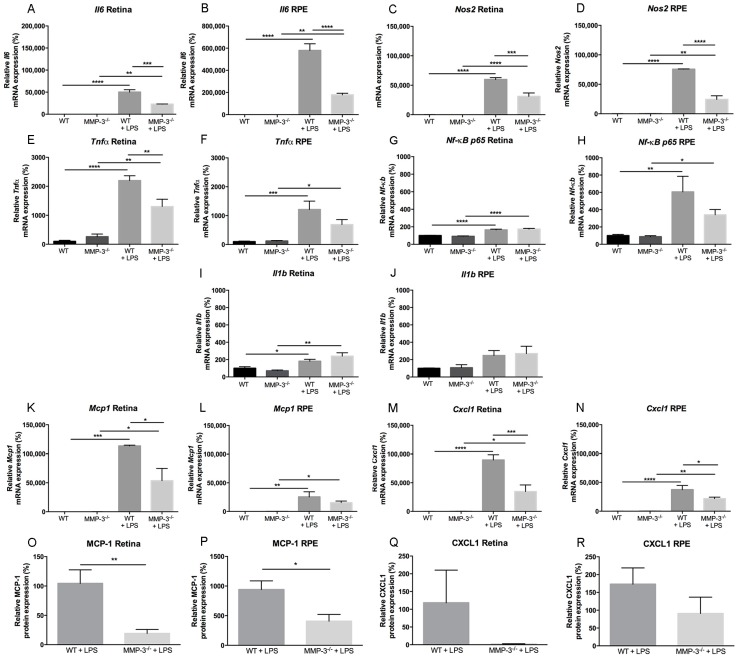
MMP-3 deficiency prevents strong upregulation of inflammatory-associated molecules in the retina and RPE during EIU. (**A**–**N**) The gene expression levels of interleukin 6 (*Il6*), nitric oxide synthase (*Nos2*), tumor necrosis factor alpha (*Tnfα*), nuclear factor kappa B p65 subunit (*Nf-κB p65*), *Il1b* and monocyte chemoattractant protein (*Mcp1/Ccl2*), (C-X-C motif) ligand 1 (*Cxcl1*) were performed on WT and MMP-3^−/−^ retinal and RPE samples at 0 and 16 hpi. Healthy retina and RPE samples from WT and MMP-3^−/−^ mice showed very low to undetectable mRNA levels. Most of the inflammatory genes, i.e., *Il6* (**A**,**B**), *Nos2* (**C**,**D**), *Tnfα* (**E**,**F**), *Mcp1* (**K**,**L**) and *Cxcl1* (**M**,**N**) were significantly upregulated in WT eyes subjected to EIU (16 hpi), and to a much lesser extent, in the MMP-3^−/−^ retina and RPE; *Nf-κB p65* (**G**,**H**) is predominantly upregulated in the RPE, and MMP-3 deficiency reduces its levels in the RPE, yet not significantly; *Il1b* (**I**,**J**) showed only a limited upregulation in WT and MMP-3^−/−^ retinal and RPE samples subjected to EIU, and no clear difference was revealed between WT and MMP-3^−/−^ mice after LPS. For most of the inflammatory molecules, the highest increase in mRNA levels, as well as the most pronounced difference between the WT and MMP-3^−/−^ condition, is observed in the RPE. RT-PCR data were normalized against the reference genes and were expressed as a percentage relative to 0 hpi (*n* ≥ 3); (**O**–**R**) Retinal and RPE samples from WT and MMP-3^−/−^ mice with or without LPS injection were also analyzed for the pro-inflammatory chemokines MCP-1 (**O**,**P**) and CXCL1 (**Q**,**R**) at the protein level via cytometric bead array at 16 hpi. Levels of both MCP-1 and CXCL1 are significantly upregulated after EIU in WT mice and MMP-3 deficiency clearly suppressed this augmentation, especially the MCP-1 protein levels. Data are shown as mean ± SEM, *n* ≥ 5, * *p* ≤ 0.05, ** *p* ≤ 0.01, *** *p* ≤ 0.001 and **** *p* ≤ 0.0001. Data with no indicated statistical analysis point out no significant results.

To determine whether MMP-3 deficiency may ameliorate ocular inflammatory responses induced by systemic LPS injection, and to further explore the mechanisms by which MMP-3 deficiency suppresses the previously described inflammatory processes in the EIU mouse model, real time quantitative PCR for interleukin 6 (*Il6*), *Il1b*, tumor necrosis factor alpha (*Tnfα*), nuclear factor kappa B p65 subunit (*Nf-κB p65*), monocyte chemoattractant protein (*Mcp1/Ccl2*), (C-X-C motif) ligand 1 (*Cxcl1*) and nitric oxide synthase (*Nos2*) (Figure 5A–N), and cytokine bead array (CBA) for MCP-1 and CXCL1 (Figure 5O–R) were performed on WT and MMP-3^−/−^ retinal and RPE samples at 0 and 16 hpi. Compared to WT mice, MMP-3 deficiency did not affect the basal mRNA expression patterns of any of the inflammation-associated genes in the retina or RPE (Figure 5A–N). Notably, mRNA levels of these inflammatory molecules were very low to undetectable. After EIU induction, the levels of all genes were significantly increased in WT and MMP-3^−/−^ mice in both the retina and the RPE (Figure 5A–N), except for *Il1b* in the RPE, which only showed a small increment in both genotypes (Figure 5J). Moreover, not all inflammatory molecules were equally augmented in WT mice with EIU, including differences between retina and RPE samples. Indeed, while *Il1b* seems barely upregulated after EIU in the mouse retina and RPE, *Mcp1* and *Cxcl1* are the most affected genes during EIU, especially in the retina. In addition, *Il6*, *Nos2* and *Tnfα* are clearly upregulated after LPS injection. Furthermore, this increment in *Il6* and *Nos2* levels was more pronounced in the RPE than in the retina, similar as for *Nf-κB p65*. Interestingly, compared to WT mice with EIU, MMP-3 deficiency (significantly) reduced the retinal and RPE mRNA levels of most of these inflammatory molecules, except for *Il1b* in both tissues and *Nf-κB p65* in the retina, which are expressed only at low levels in these tissues during EIU. In addition, while protein levels of MCP-1 and CXCL1 were undetectable in retina and RPE of both MMP-3^−/−^ and WT eyes in baseline conditions, they were increasingly observed in the two genotypes at 16 hpi (Figure 5O–R). Strikingly, MMP-3 deficiency clearly reduced the level of both chemokines in retina and RPE samples (Figure 5O–R). Indeed, MCP-1 levels were significantly reduced by 81% (*p* ≤ 0.01) in the retina and by 56% (*p* ≤ 0.05) in the RPE of MMP-3^−/−^ mice in comparison to WT animals. Accordingly, MMP-3 deficiency reduced CXCL1 levels by 98% in the retina and by 47% in the RPE, yet variability in samples prevented to reach statistical significance. Overall, our data reveal that MMP-3 deficiency clearly reduces the inflammatory response after EIU in the retina and RPE.

### 2.6. Impact of MMP-3 on the Adhesion Molecule Intercellular Adhesion Molecules (ICAM)-1 after EIU in the Retina and RPE

**Figure 6:**
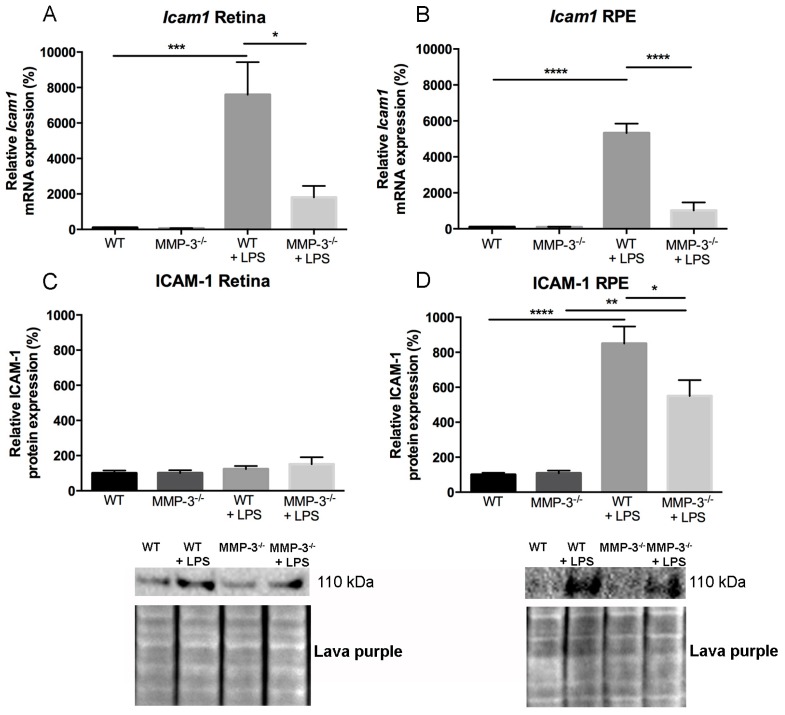
The effect of MMP-3 deficiency on ICAM-1 mRNA and protein levels in the retina and RPE during EIU. (**A**,**B**) Real time-PCR data revealed significantly elevated levels of *Icam1* at 16 h after EIU induction in the posterior eye segment of WT eyes. This augmentation was significantly reduced in retinal and RPE samples of MMP-3^−/−^ mice as compared to WT animals. Data were normalized against the reference genes and were expressed as a percentage relative to 0 hpi (*n* ≥ 3); (**C**) The protein level of ICAM-1 in the retina only showed a slight increase after EIU, and no clear difference between WT and MMP-3^−/−^ mice after LPS; (**D**) However, ICAM-1 in the RPE was significantly increased at 16 hpi in both genotypes. Interestingly, MMP-3 deficiency reduced ICAM-1 levels after LPS. Lava purple staining served as a loading control. Western blot data were normalized against Lava purple total protein stain and expressed as a percentage relative to 0 hpi. All data are shown as mean ± SEM, *n* ≥ 5, * *p* ≤ 0.05, ** *p* ≤ 0.01, *** *p* ≤ 0.001, **** *p* ≤ 0.0001. Data with no indicated statistical analysis point out no significant results.

Next to the cytokines and chemokines described above, the adhesion molecule ICAM-1 is also a well-known and important modulator of leukocyte recruitment and adhesion during inflammation in the retina and RPE [2,44,45]. To investigate whether ICAM-1 levels are influenced by MMP-3 in the EIU model and thereby contribute to the observed reduction in adherent leukocytes/infiltrating leukocytes, changes in the gene and protein expression levels of ICAM-1 were explored in control and LPS-treated WT and MMP-3^−/−^ eyes. In the healthy WT and MMP-3^−/−^ retina and RPE, *Icam1* mRNA levels were barely detectable (Figure 6A,B). However, EIU induction in WT mice resulted in a significant upregulation of *Icam1* mRNA in the retina and the RPE. Importantly, MMP-3 deficiency clearly attenuated the gene expression levels of *Icam-1*, since a reduction of 76% of *Icam1* was observed in both the retina (*p* ≤ 0.05) and RPE (*p* ≤ 0.0001) of MMP-3^−/−^ mice as compared to WT mice. To confirm the effect of MMP-3 deficiency on the reduction of *Icam-1* expression, additional Western blot experiments were performed to investigate ICAM-1 at the protein level, both in retina and RPE samples (Figure 6C,D). In the retinas of mice with EIU, only a limited upregulation of ICAM-1 protein expression was observed, and the attenuating effect of MMP-3 deficiency was no longer obvious. However, in the RPE, ICAM-1 protein levels were significantly increased after EIU in both WT and MMP-3^−/−^ eyes. Importantly, MMP-3 deficiency clearly reduced the levels of ICAM-1 during EIU, more specifically by 35% as compared with WT RPE samples. As such, after EIU induction, our data suggest that MMP-3 affects ICAM-1 expression at the posterior part of the eye, especially at the RPE.

### 2.7. MMP-3 Deficiency Does Not Influence Zona Occludens 1 (ZO-1) Levels at the Inner or Outer Blood–Retinal Barrier (BRB) during EIU

**Figure 7:**
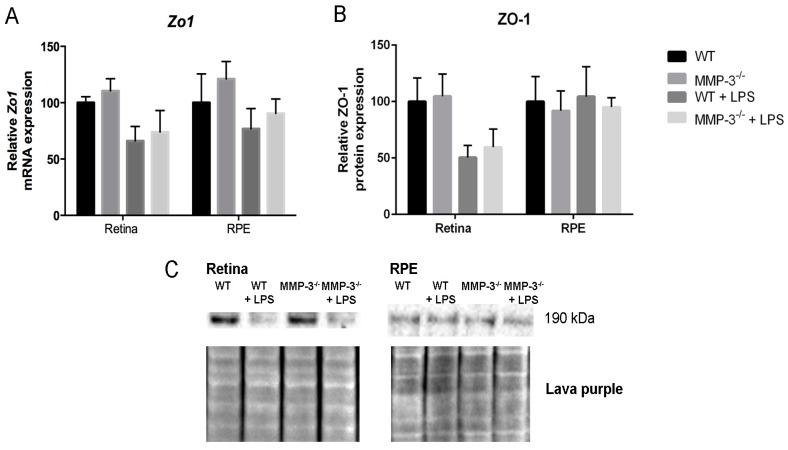
The impact of MMP-3 deficiency on ZO-1 in the retina and RPE during EIU. (**A**) Real time-PCR data showed a clear reduction, although not significant, in *Zo1* mRNA levels in the retina of WT and MMP-3^−/−^ mice after EIU induction, which was not observed in the RPE; (**B**,**C**) Additional Western blot analysis of ZO-1 in the retina and RPE confirmed these data. Lava purple staining served as a loading control. Western blot data were normalized against Lava purple total protein stain and expressed as a percentage relative to 0 hpi. (**A**–**C**) MMP-3 deficiency did not affect ZO-1 levels during EIU at the posterior eye segment. Data are shown as mean ± SEM, *n* ≥ 5.

To investigate whether systemically-induced ocular inflammation affects the levels of the tight junction protein zona occludens 1 (ZO-1) at the inner (endothelial cells) and outer (RPE cells) BRB, and to evaluate the influence of MMP-3 deficiency on this protein, ZO-1 expression was studied at the transcriptional (Figure 7A) and translational (Figure 7B,C) level at 16 hpi in WT and MMP-3^−/−^ RPE and retinal samples. LPS administration reduced ZO-1 mRNA and protein levels in the retina of WT mice, more than in the RPE (*p* = NS). ZO-1 levels were decreased to a similar extent in MMP-3^−/−^ retina and RPE samples. Thus, ZO-1 levels seem reduced in particular at the inner BRB during EIU and MMP-3 deficiency did not affect ZO-1 levels in the posterior segment of the eye during EIU.

## 3. Discussion

Although matrix metalloproteinase-3 (MMP-3 or stromelysin-1) has been described to importantly contribute to several neuroinflammatory processes, such as leukocyte adhesion and infiltration, micro/macroglial activation, release/activation of cytokines and free radicals in the brain and spinal cord and at its barriers [11,16,31,46,47,48], we aimed to investigate the role of this protease in acute inflammation in the eye, with a focus on the retina, the RPE and the blood–retinal barrier (BRB). These studies are of fundamental significance to increase our understanding of ocular inflammation, as inflammatory processes and BRB dysfunctions are strongly implicated in the pathogenesis of highly prevalent ocular diseases such as diabetic retinopathy, age-related macular degeneration (AMD), Behçet’s disease, retinitis pigmentosa and posterior uveitis [4,49,50]. Therefore, a potential common therapeutic strategy in combatting these disorders will presumably link with the regulation of inflammatory processes [50].

In this study, we provide important evidence for the contribution of MMP-3 to acute retinal inflammation, using a mouse model of endotoxin-induced uveitis (EIU). At first, before induction of EIU, retinal morphology and visual acuity were investigated in MMP-3 deficient and WT mice. No difference in both measurements was observed between the genotypes, meaning that MMP-3 is seemingly dispensable for proper formation of retinal layers and normal visual functioning. However, we cannot rule out any proteolytic redundancy or enzymatic compensation during development in the MMP-3^−/−^ mice. Indeed, the lack of clear phenotypes in MMP mutant mice has been attributed to, amongst others, the overlapping substrates between MMPs or the upregulation of other MMPs within this large family [51].

We confirmed the presence of MMP-3 in the healthy retina, more specifically in Müller glia end feet and their fibers located in the inner retina, as previously described [52]. EIU promoted MMP-3 mRNA and protein levels to some extent in the retina, but dramatically induced its expression in the RPE. Notably, our data also demonstrated an increased MMP-3 protein secretion into the vitreous, suggesting a possible translocation of MMP-3 from the inner retina to the vitreous after LPS. Remarkably, especially pro MMP-3 levels were found to be upregulated in the RPE and vitreous after LPS treatment. MMPs, and also MMP-3, are classically activated via proteolytic cleavage of the pro-peptide, which results in opening of the cysteine-to-zinc switch. After activation, mature MMP-3 exerts its function and will soon be captured, presumably by tissue inhibitors of metalloproteinases (TIMPs), and possibly be proteolytically degraded and physically cleared to maintain tissue homeostasis [52,53]. Alternatively, MMP-3 can also be activated via non-proteolytic compounds such as sulfydryl-reactive agents and reactive oxygen species (ROS), which react with the sulfydryl groups and inactivate the cysteine without pro-peptide removal [53,54]. As such, pro MMP-3 might, after activation by ROS, which is known to be quickly induced by LPS and shown to be involved in ocular inflammatory disorders [55,56,57], exert its functions and contribute to acute inflammatory processes. Thus far, however, there are no data that could explain the role or function of MMP-3 in the vitreous during ocular inflammation. Nevertheless, our findings in retinal and RPE tissue correspond to the observed increment in MMP-3 levels during the early neuroinflammatory phase after brain and spinal cord injury [16,58].

Increased transcription (and translation) of MMP-3 during EIU can be elicited via inflammatory cytokines, as the *Mmp3* gene contains binding sites for the transcription factors AP-1 and NF-κB in its promoter region. Indeed, many of the responses to the pro-inflammatory cytokine TNF-α require de novo gene expression regulated by these transcription factors [27,28,29,59]. As such, our data suggest that the upregulated levels of TNF-α during EIU might induce expression of (the p65 subunit of) NF-κB, especially at the RPE, thereby mediating production of MMP-3 in this tissue. The endotoxin LPS is known to activate the inflammatory cascade via Toll-like receptor 4 (TLR4), present on immune cells, including neutrophils, macrophages and lymphocytes, but also on epithelial cells, including choroid plexus epithelial (CPE) cells, whereby the stimulation of TLR4 by LPS causes the release of pro-inflammatory cytokines, including IL-6 and TNF-α, necessary to activate potent immune responses, such as the adhesion and activation of leukocytes [60,61,62,63]. Previous findings also indicate the presence of TLR4 receptors on human RPE cells, which upon ligand binding lead to nuclear translocation of NF-κB and subsequently, the activation of pro-inflammatory cytokines [64]. Interestingly, an increase in MMP-3 levels has already been demonstrated in cultured human RPE cells after oxidative stress [65] and in the presence of TNF-α [34,35], as well as in RPE/choroid samples of AMD patients and of old mice compared to those of young animals [36,66]. Moreover, treatment of porcine CPE cells with TNF-α or the *Streptococcus suis* bacterium also induced a clear upregulation in *Mmp3* gene expression levels [2,67]. Finally, it has also been described that intracerebral injection of LPS stimulates the expression of MMP-3 in brain tissue [21] and intracerebroventricular injection of Aβ1-42 oligomers, a validated Alzheimer’s disease mouse model characterized by a strong inflammatory response at the CPE and associated disruption of the BCSFB, revealed a marked upregulation of MMP-3 mRNA and protein expression in CPE cells [14]. Overall, our data show a prominent upregulation of MMP-3 during acute neuroinflammatory responses at the posterior part of the eye, more specifically at the RPE, suggesting an important role for this protease in modulating ocular inflammation.

Consistent with previous mouse EIU studies, the number of leukocytes that firmly adhered to the retinal vasculature in WT or vehicle-treated eyes was significantly increased during EIU [4,5]. Importantly, our study established, using genetic and pharmacological approaches, that MMP-3 is involved in leukocyte adhesion during acute inflammation in the eye. As such, our data disclosed a clearly reduced leukostasis in MMP-3 deficient mice compared to WT animals. Moreover, a trend towards a diminished number of infiltrating vitreous leukocytes at the optic nerve head, a known site for early infiltration, was observed in MMP-3^−/−^ mice, as compared to WT eyes, after induction of endotoxemia. Administration of the pharmacological MMP-3 inhibitor NNGH even resulted in a more pronounced decrease in leukocyte adhesion. In addition, the attenuated hypothermic response was also more explicit after pharmacological than genetic inactivation of MMP-3 after systemic LPS injection. This small discrepancy in body temperature and leukocyte adhesion between MMP-3^−/−^ and NNGH inhibitor-treated mice might be due to the implication of other MMPs, beside MMP-3, in LPS-associated inflammatory processes. Indeed, MMPs are well known to modulate LPS-induced inflammatory responses, such as leukocyte rolling and adhesion, neutrophil influx and the activation of cytokines, and might as such contribute to the hypothermic response. Although the precise mechanisms underlying hypothermia are not fully understood, many inflammatory mediators seem to contribute to this complex process [68,69,70]. Importantly, several expression studies have observed upregulation of multiple MMPs, including MMP-3, after an LPS challenge in different cell types and organs, and inhibition of MMPs via broad-spectrum MMP inhibitors clearly showed protection against endotoxic shock [13,71,72]. Furthermore, MMP-7, -8, -12 and -13 deficient mice were also previously reported to show protection against death and hypothermia in the systemic LPS mouse model [13,73,74,75]. Although NNGH is a potent inhibitor of MMP-3, it also blocks MMP-1, MMP-2, MMP-7, MMP-8, MMP-9, MMP-10, MMP-12, MMP-13, MMP-14, and MMP-20 activity. Treatment of MMP-3^−/−^ mice with NNGH resulted in a reduced hypothermic response as compared to vehicle-injected MMP-3^−/−^ animals. Together, these findings suggest that absent/decreased MMP-3 levels during EIU exert protective and anti-inflammatory effects. Moreover, these results also indicate that one or several of the above mentioned MMPs may provide an additive effect to MMP-3 on the hypothermic response after endotoxin-induced acute inflammation, and thus probably also to leukocyte adhesion/infiltration in the eye.

Several reports demonstrated an increase of IL-6 in the retina and RPE after systemic LPS injection, where it is assumed to play an essential role in the development of EIU through activation of microglia and astrocytes [7,76,77,78,79,80]. While *Il6* mRNA levels was barely detected in the healthy retina and RPE in this study, *Il6* levels were strongly upregulated in the retina and RPE of EIU mice. Importantly, MMP-3 deficiency significantly suppressed this increase in *Il6 gene expression* during EIU. During the initial stages of inflammation, both the endothelium and epithelium in the CNS are stimulated by locally produced cytokines, such as IL-6, which in turn activate/increase chemokines and adhesion molecules [2,81]. Indeed, as confirmed in our study, chemokines, such as MCP-1 and CXCL1, but also the adhesion molecule ICAM-1, are upregulated during EIU, thereby exerting important roles in leukocyte recruitment and adhesion during ocular inflammation [45,80,82]. Importantly, our results clearly disclosed a reduction of MCP-1 and CXCL1 mRNA and protein levels in both retinal and RPE samples of MMP-3 deficient mice during EIU. Our data also demonstrated low *Icam1* mRNA expression in the healthy retina and RPE, and an elevation of *Icam1* levels in the inflamed retina and RPE/choroid complex. Western blot data revealed a similar increase in ICAM-1 protein levels in the RPE during EIU, yet this upregulation was reduced in retinal samples, indicating that ICAM-1 especially plays a pivotal role in regulating leukocyte adhesion at the RPE during retinal injury. These findings further support the importance of the RPE as gateway for monocyte trafficking to the retina, similar as observed for the epithelial barriers in the brain. As such, it was recently reported that monocytes would accumulate first in the RPE before entering the retina [83]. Previous studies reported that shortly after systemic LPS injection, ICAM-1, as well as its interaction partners, including leukocyte counter receptors lymphocyte function-associated antigen 1 (LFA-1) and macrophage antigen 1 (Mac-1), are upregulated in blood vessels of the retina and RPE/choroid complex, thereby enhancing leukocyte recruitment and adhesion [5,45,50]. Interestingly, our data indicated that MMP-3 highly influences ICAM-1 levels at the RPE/choroid complex, but to a lesser extent in the retina. During CNS inflammation, NOS2 (or iNOS) is a well-established source of NO, which is an important second messenger that regulates many physiological and pathological events, including inflammatory processes. NO seems to be pro-inflammatory in EIU because increased NOS2 levels are associated with inflammatory conditions and inhibition of NOS2 has been shown to limit the pathogenesis of EIU [10,84]. Moreover, endotoxin and cytokine stimulation in the EIU model was reported to induce expression of NOS2 in Müller glia and RPE cells [84]. Interestingly in our study, MMP-3 deficiency resulted in clearly attenuated levels of *Nos2* in the retina, but especially in the RPE, again indicating an involvement of MMP-3 in the inflammatory response in the EIU model.

Overall, we hypothesize the existence of a feedback loop during EIU, in which activation of TLR4 by LPS stimulates expression/activation of pro-inflammatory cytokines, amongst others TNF-α, which in turn activates transcription factors such as NF-κB, in this case most prominently at the RPE. Translocation of NF-κB to the nucleus is known to activate transcription of several genes, including *Mmp3*, *Icam1*, *Il6*, *Mcp1* and *Nos2* [29,50,59,79,80]. Intriguingly, these molecules are prominently present in the RPE/choroid tissue after systemic LPS administration. Alternatively, also activation of the MAP kinase P38 or ERK pathway after induction of endotoxemia can possibly trigger cytokine-induced MMP-3 transcription/translation [71,85,86,87,88]. When then MMP-3 becomes activated, for instance by ROS, it might in turn trigger the release of TNF-α and/or activation of pro TNF-α [89,90], thereby again contributing to the inflammatory responses and cascades. In addition, by cleaving the protease-activated receptor-1 into an active ligand, mature MMP-3 can contribute to microglia activation, and thus stimulate expression of cytokines and other inflammatory molecules [31,83]. Finally, we cannot exclude the contribution of other pathways, which are known to be affected by MMP-3, in LPS-induced inflammation in the eye. Overall, based on the above, it is clear that when MMP-3 production/activation is prevented, this will cause a reduction in the level of inflammation-associated molecules. Remarkably, papers describing the effect of MMP-3 on inflammatory molecules and processes in the CNS are rather poor. It has been reported that inhibition of MMP-3 significantly suppresses gene expression levels of *Nos2*, *Il6*, *Il1b* and *Il1Ra* in cultured microglial cells treated with LPS [32]. We also observed a strongly diminished expression of *NOS2* and *Il6* in MMP-3^−/−^ mice as compared to WT mice, yet no effect on *Il1b* was found, probably due to its very low expression levels in EIU WT mice. In addition, it is known that during acute inflammation, MMP-3 facilitates neutrophil infiltration by disrupting the BBB and BSCB, probably via degradation of basal lamina components and tight junction proteins including ZO-1 [11,16,33]. Previous reports suggest that ZO-1 might play a major role in maintaining the BRB [91,92]. However, until now, studies on the role of ZO-1 at the inner and outer BRB in mice after LPS challenge are lacking, as well as the influence of MMP-3 on ZO-1 levels during ocular inflammation. We observed a decrease in ZO-1 levels at 24 h post systemic LPS administration in both genotypes in the retina, but to a lesser extent in the RPE, indicating that ZO-1 expression at the RPE is probably not affected by systemic LPS exposure. These data are line with previous findings in rats that showed a significant decrease in retinal levels of ZO-1, leading to an increase of inner BRB permeability, after systemic LPS administration [93], while incubation of cultured rat RPE cells with LPS in vitro had no effect on RPE tight junctions [94]. Of note, MMP-3 deficiency did not affect ZO-1 levels at the retina or RPE during EIU, suggesting that MMP-3 is not involved in disruption of ZO-1 at the BRB. However, future experiments are needed to further clarify a role for MMP-3 in degradation of tight junction proteins and basal lamina components at the inner and outer BRB during ocular inflammation.

In summary, our data provide the first evidence of MMP-3 as a strong modulator of acute ocular inflammation in the posterior part of the eye. Specifically, MMP-3 levels are strongly upregulated in the inflamed eye, predominantly in the RPE, during endotoxemia. Moreover, at the cellular level, MMP-3 deficiency reduces leukocyte recruitment to the retina and vitreous cavity, and, at the molecular level, MMP-3 clearly suppresses the upregulation of inflammation-related molecules. Thus, although additional studies are essential to gain more insight in the exact MMP-3 working mechanisms during ocular inflammation, these findings further support the hypothesis that MMP-3 is importantly involved in neuroinflammatory processes, and might therefore serve as a potential therapeutic target for the development of ocular anti-inflammatory strategies.

## 4. Materials and Methods

### 4.1. Animals

All experiments were performed using 8–12 week old C57Bl/6J wild-type (WT) and MMP-3 deficient (MMP-3^−/−^) mice (Mudgett, Merck Research Labs, Rahway, NJ, USA). All animals were housed under standard laboratory conditions according to a normal day/night rhythm with ad libitum access to food and water. Genotyping was performed on tail DNA using the following primers for WT DNA: 5′-aacatggagactttgtccctt-3′ as forward and 5′-cagtgacatcctctgtcc-3′ as reverse primer, and for MMP-3^−/−^ DNA: 5′-atactttctcggcaggag-3′ as forward and 5′-tgaatgaactgcaggacgag -3′ as reverse primer. All animal experiments were approved by the Institutional Ethical Committee of KU Leuven and are in strict accordance with the European Communities Council Directive of 22 September 2010 (2010/63/EU). Of note, a recently published study indicated a phenotypic interference of an inactivating passenger mutation in the *Casp11* gene within this MMP-3^−/−^ mouse line [95]. Whether this additional mutation affects the currently reported findings remains to be investigated. However, to confirm our data obtained in MMP-3^−/−^ animals, we also used a pharmacological inhibitor of MMP-3 in WT mice. Thereto, 100 µL of the MMP-3 inhibitor *N*-Isobutyl-*N*-(4-methoxyphenylsulfonyl)-glycyl hydroxamic acid (NNGH) was administered via intraperitoneal (ip) injection at a concentration of 1.6 µg/µL (in 5% DMSO, 95% PBS), 15 min before and at 6 and 14 h after LPS treatment (hours post LPS injection, hpi).

### 4.2. Endotoxemia Model

Both female and male mice received a single ip injection of lipopolysaccharides (LPS) from *Salmonella enterica* serotype *abortus equi* (Sigma-Aldrich, St. Louis, MO, USA), diluted in Dulbecco’s phosphate buffered saline (dPBS) at a dose of 12.5 mg/kg body weight. This dose was selected after titration experiments and results in 80% mortality (LD80) in WT mice. Rectal temperature was measured three times a day after LPS challenge. Mice that did not respond to LPS were omitted from the experiments.

### 4.3. Quantitative Real-Time PCR

Animals were sacrificed at 0 (no LPS injection), 8, 16 and 24 hpi for WT mice and after 0 and 16 hpi for MMP-3^−/−^ mice (*n* ≥ 3), and total RNA was extracted from both retina and RPE/choroid/sclera (further on indicated as RPE) using Tri-reagent (Sigma-Aldrich) and purified by NucleoSpin RNA clean-up Isolation Kit (MACHEREY-NAGEL, Düren, Germany). RNA was reverse transcribed to cDNA using Superscript III reverse transcriptase (Invitrogen, Ghent, Belgium) with poly-dT primers. Real-time quantitative PCR for *Mmp3* at 0, 8, 16 and 24 hpi and for zona occludens 1 (*Zo1*), intercellular adhesion molecule 1 (*Icam1*), nitric oxide synthase 2 (*Nos2* or *iNos*), interleukin 6 (*Il6*), *Il1b*, tumor necrosis factor (*Tnfα*), nuclear factor kappa B p65 subunit (*Nf-κB p65*), monocyte chemoattractant protein (*Mcp1/Ccl2*), and (C-X-C motif) ligand 1 (*Cxcl1*) at 0 and 16 hpi was performed using Taqman assays (Thermo Scientific, Ghent, Belgium) and a StepOne Plus apparatus (Applied Biosystems, Thermo Scientific). Thermal cycle parameters were 10 min at 95 °C, followed by 40 cycles of 10 s at 95 °C and 60 s at 60 °C. All samples were run in duplicate. Using geNorm software (qBase, available online: https://genorm.cmgg.be/), Tyrosine 3-monooxygenase/tryptophan 5-monooxygenase activation protein zeta (*Ywhaz*), 18S Ribosomal RNA (*Rn18s1*) and β-glucuronidase (*Gusb*) were selected as reference genes. Expression levels were analyzed using qBase software, an advanced quantification tool based on the ΔΔ*C*_t_ quantification method, and normalized against the geometric mean of the three reference genes. All primers and probes are listed in Table 1, and constructed by Integrated DNA Technologies (IDT).

### 4.4. Western Blotting

Retina and RPE samples from both genotypes were isolated and immediately transferred into ice cold 100 µL lysis buffer (50 mM Tris, 10 mM CaCl_2_, 150 mM NaCl, 0.05% Brij 35, and 1% Triton-X-100, pH 7.5), supplemented with protease inhibitor (Roche Applied Science, Mannheim, Germany), one mini tablet per 10 mL of lysis buffer). Samples were homogenized by sonication for 1 min and centrifuged for 10 min at 18,000× *g*. Protein concentrations were determined according to the Bradford method (Bio-Rad, Temse, Belgium). Proteins from retinal, RPE and vitreous samples were separated on 4%–12% NuPage^®^ Bis-Tris gels (Invitrogen) and transferred using the TransBlot^®^ TurboTM Transfer System (Bio-Rad) onto PVDF or nitrocellulose membranes (Bio-Rad). The membranes were blocked for 2 h in 5% commercial blocking powder (ECL™ Blocking agent, GE Healthcare, Diegem, Belgium) and incubated overnight at 4 °C with antibodies to MMP-3 (1/200, Ab52915, Abcam, Cambridge, UK), ICAM-1 (1/60, SC-1511, Santa Cruz, CA, USA) and ZO-1 (1/80, ab59720, Abcam). After incubation with horseradish peroxidase (HRP)-labeled secondary antibodies, the membranes were incubated in Supersignal^®^ West Dura Extended Duration Substrate solution (Thermo Scientific). The ChemiDoc MP Imager (Bio-Rad) was used to measure optical densities of the protein bands, which were normalized against Lava purple total protein stain (Gelcompany, San Francisco, CA, USA). Western blot experiments were performed on retinal/RPE/vitreous samples from at minimum 5 mice per genotype and were repeated at least twice.

### 4.5. Immunohistochemistry (IHC)

Mice were deeply anesthetized (ip of 30 mg/kg sodium pentobarbital, Nembutal, Ceva, Brussels, Belgium) and transcardially perfused with 4% phosphate buffered paraformaldehyde (PFA), and sagittal paraffin- or cryosections (10 µm) of the eye were made. General eye morphology was studied using hematoxylin and eosin (H&E) staining. To determine regional and cellular expression of MMP-3, immunofluorescent stainings were performed. Briefly, upon antigen retrieval method with heated citrate buffer (10 mM citric acid, 0.05% Tween 20, pH 6.0), endogenous peroxidases were blocked by incubation in 0.3% H_2_O_2_ (in methanol) for 20 min, and subsequently incubated with 20% pre-immune serum before overnight incubation with the primary antibody MMP-3 (1/200, Ab52915, Abcam). Next, Alexa Fluor-conjugated secondary antibodies (Invitrogen) or the TSA^TM^ FITC kit (PerkinElmer, Waltham, MA, USA) were applied as previously published [96,97] and stained sections were examined and photographed using an Olympus FV1000 confocal microscope (Olympus, Berchem, Belgium).

### 4.6. Multiplex Cytokine Assay

The BD cytokine bead array (CBA) kit (BD Biosciences, San Jose, CA, USA) was used to quantitatively measure chemokines CXCL1 (or KC) and MCP-1, in the retina and RPE of WT and MMP-3^−/−^ mice subjected to EIU. Samples were prepared as described above for Western blot, and 25 µL of mouse cytokine standards and samples were used to enable fluorescence detection of the antibody-coated beads, according to the manufacturer’s instructions. Flow cytometry was performed using the BD FACS canto HTS (BD Biosciences). CBA results were analyzed using FCAP ArrayTM software (BD Biosciences).

### 4.7. Retinal Leukocyte Adhesion Quantification

Mice were deeply anaesthetized (ip injection of 30 mg/kg sodium pentobarbital, Nembutal, Ceva) at 24 hpi and were transcardially perfused with NaCl 0.9% + heparin (71 mg/L) during 5 min (2–3 mL/min) to remove erythrocytes and non-adherent leukocytes. Next, the animals were perfused with 25 mL of FITC-Concanavalin A lectin (FITC-ConA; 40 µg/mL in PBS 1X, pH 7.5) at a flow rate of 10 mL/min, followed by removal of unbound FITC-ConA with NaCl-heparin during 5 min (2–3 mL/min). After cervical dislocation, the eyes were isolated, incubated overnight in 1% PFA and flatmounted. Each retina was visualized under a Leica DM IL LED microscope (Leica, Wetzlar, Germany) and the total number of FITC-ConA stained adherent leukocytes was counted by a blinded investigator, in all arterioles and venules. Representative pictures were taken using an Olympus FV1000 confocal microscope.

### 4.8. Optical Coherence Tomography

To assess thickness of the retinal layers and retinal morphology in general, and to evaluate inflammatory leukocyte infiltration into the vitreous cavity at 24 hpi in vivo, a spectral domain optical coherence tomography (SD-OCT) system (Envisu R2210, Bioptigen, Morrisville, NC, USA) was used. After anesthetizing the mouse via ip injection of 75 mg/kg body weight ketamine (Anesketin, Eurovet, Bladel, The Netherlands) and 1 mg/kg medetomidine (Domitor, Pfizer, NY, USA), topical application of 0.5% tropicamide (0.5% Tropicol, Thea Pharma, Wetteren, Belgium) was used to dilute the pupils. Next, SD-OCT was accomplished via 100 serial B-scan lines with each line consisting of 1000 A-scans, in a 1.4 mm × 1.4 mm field. Afterwards, ip injection of atipamezol (1 mg/kg, Antisedan, Pfizer) was applied to reverse the anesthesia, followed by antibiotic ointment to the eye (tobramycin 3 mg/g, Tobrex, Alcon, Puurs, Belgium) To evaluate retinal morphology, total retinal thickness and thickness of the ganglion cell complex (GCC), which comprises the nerve fiber layer (NFL), ganglion cell layer (GCL) and the inner plexiform layer (IPL), of WT and MMP-3^−/−^ mice were analyzed using InVivoVue Diver 2.2 software (Bioptigen). Cellular infiltrates were defined as hyperreflective dots in the vitreous cavity that were larger and of greater density than background noise [98]. Per eye, these infiltrating leukocytes were measured manually over a distance of 500 µm around the optic nerve head on 5 B-scan images: 1 B-scan image centered at the optic nerve head, and 4 images at 50 µm intervals from the optic nerve head (2 anterior, 2 posterior) (see also Figure 4D). Values of these 5 images were summed up to yield a representative number of infiltrating leukocytes for each retina.

### 4.9. Optokinetic Tracking Response

The optokinetic tracking response was performed in photopic conditions in a virtual-reality chamber (OptoMotry, Cerebral Mechanics, Medicine Hat, AB, Canada), as previously described [99,100,101]. Briefly, the mouse was situated on a platform in the middle of a virtual cylinder, that consist of a vertical sine wave grating projecting on four computer screens into a closed box. Real-time video feedback is provided via a video camera placed above the mouse. Visual acuity of each eye was determined using a staircase procedure, composed of randomly different spatial frequencies (100% contrast, 12° per second speed). A blinded experimenter determined the maximum spatial frequency at which optokinetic tracking was seen.

### 4.10. Statistical Analysis

All values are represented as mean ± SEM. Statistical analyses were performed with GraphPad Prism 6 (GraphPad Software, San Diego, CA, USA) using student’s *t*-test, one or two way-ANOVA, with Tukey’s multiple comparisons or a Bonferroni test as post-hoc tests. For all statistical tests, a *p*-value of 0.05 was considered statistically significant.

## Figures and Tables

**Table 1:** Primer and probe sequences of the different genes used in RT-PCR.

Gene	Forward Primer	Reverse Primer	Probe
*Ywhaz*	cgtacctacaatgcctccatc	catcctgctgtccattgctta	tccttgattttgcctcagctccca
*Rn18s1*	cgtacctacaatgcctccatc	agaattgcaacagctcgattg	cagcccctgatgagaatgtaccagc
*Gusb*	gacccaagataccgacatgag	cagcttgctaatgtcagcct	aatcccattcacccacacaactgc
*Mmp3*	gatgaacgatggacagaggatg	tgtggaggacttgtagactgg	tggttgctgctcatgaacttggc
*Il6*	tccttagccactccttctgt	agccagagtccttcagaga	cctaccccaatttccaatgctctcct
*Il1b*	ctcttgttgatgtgctgctg	gacctgttctttgaagttgacg	ttccaaacctttgacctgggctgt
*Tnfα*	tctttagatccatgccgttg	agaccctcacactcagatca	ccacgtcgtagcaaaccaccaagt
*Nf-κBp65*	ccctctgttttggttgctcta	tgaacactgctttgactcact	atgggccatctgttgacagtggt
*Mcp1*	aactacagcttctttgggaca	catccacgtgttggctca	actcacctgctgctactcattcacc
*Cxcl1*	gtgccatcagagcagtct	ccaaaccgaagtcatagcca	aggtgtccccaagtaacggagaaag
*Icam1*	ggtccttgcctacttgctg	ctgtgctttgagaactgtgg	ccgctaccatcaccgtgtattcgtt
*Nos2*	cacttctgctccaaatccaac	gactgagctgttagagacactt	tgaacaagacccaagcgtgaggag
*Zo1*	gcaactacagtatgaccatcc	aatgaataatatcagcaccatgcc	ctgtccctgtgagtccttcagctg
*Rplp2*	gtcatcgctcagggtgttg	gactcctccttcttctcatctttc	ctgtggctgtttctgctgccc
*Hprt*	cgtgattagcgatgatgaacc	catgacatctcgagcaagtct	cctcatggactgattatggacaggactga

## Abbreviations

CNS	central nervous system
LPS	lipopolysaccharide
MMP-3	matrix metalloproteinase 3
WT	wild-type
RPE	retinal pigment epithelium
ip	intraperitoneal
EIU	endotoxin-induced uveitis
BRB	blood–retinal barrier
BBB	blood–brain barrier
BCSFB	blood–cerebrospinal fluid barrier
BSCB	blood–spinal cord barrier
ICAM-1	intercellular adhesion molecule 1
IL/*Il*	interleukin
NOS2	nitric oxide synthase 2
MCP-1	monocyte chemoattractant protein 1
CXCL1	(C-X-C motif) ligand 1
TNF-α	tumor necrosis factor α
NF-κB p65	nuclear factor kappa B p65 subunit
NNGH	*N*-Isobutyl-*N*-(4-methoxyphenylsulfonyl)-glycyl Hydroxamic Acid

## References

[1] Caspi R.R. (2010). A look at autoimmunity and inflammation in the eye. J. Clin. Investig..

[2] Crane I.J., Liversidge J. (2008). Mechanisms of leukocyte migration across the blood-retina barrier. Semin. Immunopathol..

[3] Yadav U.C., Ramana K.V. (2013). Endotoxin-induced uveitis in rodents. Methods Mol. Biol..

[4] Kanda A., Noda K., Oike Y., Ishida S. (2012). Angiopoietin-like protein 2 mediates endotoxin-induced acute inflammation in the eye. Lab. Investig. J. Tech. Methods Pathol..

[5] Suzuki M., Noda K., Kubota S., Hirasawa M., Ozawa Y., Tsubota K., Mizuki N., Ishida S. (2010). Eicosapentaenoic acid suppresses ocular inflammation in endotoxin-induced uveitis. Mol. Vis..

[6] Tuaillon N., Shen D.F., Berger R.B., Lu B., Rollins B.J., Chan C.C. (2002). Mcp-1 expression in endotoxin-induced uveitis. Investig. Ophthalmol. Vis. Sci..

[7] Hoekzema R., Verhagen C., van Haren M., Kijlstra A. (1992). Endotoxin-induced uveitis in the rat. The significance of intraocular interleukin-6. Ophthalmol. Vis. Sci..

[8] Koizumi K., Poulaki V., Doehmen S., Welsandt G., Radetzky S., Lappas A., Kociok N., Kirchhof B., Joussen A.M. (2003). Contribution of TNF-α to leukocyte adhesion, vascular leakage, and apoptotic cell death in endotoxin-induced uveitis in vivo. Ophthalmol. Vis. Sci..

[9] Satofuka S., Ichihara A., Nagai N., Yamashiro K., Koto T., Shinoda H., Noda K., Ozawa Y., Inoue M., Tsubota K. (2006). Suppression of ocular inflammation in endotoxin-induced uveitis by inhibiting nonproteolytic activation of prorenin. Ophthalmol. Vis. Sci..

[10] Zhang W., Baban B., Rojas M., Tofigh S., Virmani S.K., Patel C., Behzadian M.A., Romero M.J., Caldwell R.W., Caldwell R.B. (2009). Arginase activity mediates retinal inflammation in endotoxin-induced uveitis. Am. J. Pathol..

[11] Gurney K.J., Estrada E.Y., Rosenberg G.A. (2006). Blood-brain barrier disruption by stromelysin-1 facilitates neutrophil infiltration in neuroinflammation. Neurobiol. Dis..

[12] Yang Y., Rosenberg G.A. (2011). Blood-brain barrier breakdown in acute and chronic cerebrovascular disease. Stroke J. Cereb. Circ..

[13] Vandenbroucke R.E., Dejonckheere E., van Lint P., Demeestere D., van Wonterghem E., Vanlaere I., Puimege L., van Hauwermeiren F., de Rycke R., Mc Guire C. (2012). Matrix metalloprotease 8-dependent extracellular matrix cleavage at the blood-CSF barrier contributes to lethality during systemic inflammatory diseases. J. Neurosci..

[14] Brkic M., Balusu S., van Wonterghem E., Gorle N., Benilova I., Kremer A., van Hove I., Moons L., de Strooper B., Kanazir S. (2015). Amyloid β oligomers disrupt blood-CSF barrier integrity by activating matrix metalloproteinases. J. Neurosci..

[15] Wiggins-Dohlvik K., Merriman M., Shaji C.A., Alluri H., Grimsley M., Davis M.L., Smith R.W., Tharakan B. (2014). Tumor necrosis factor-α disruption of brain endothelial cell barrier is mediated through matrix metalloproteinase-9. Am. J. Surg..

[16] Lee J.Y., Choi H.Y., Ahn H.J., Ju B.G., Yune T.Y. (2014). Matrix metalloproteinase-3 promotes early blood-spinal cord barrier disruption and hemorrhage and impairs long-term neurological recovery after spinal cord injury. Am. J. Pathol..

[17] Noble L.J., Donovan F., Igarashi T., Goussev S., Werb Z. (2002). Matrix metalloproteinases limit functional recovery after spinal cord injury by modulation of early vascular events. J. Neurosci..

[18] Lakhan S.E., Kirchgessner A., Tepper D., Leonard A. (2013). Matrix metalloproteinases and blood-brain barrier disruption in acute ischemic stroke. Front. Neurol..

[19] Yang Y., Estrada E.Y., Thompson J.F., Liu W., Rosenberg G.A. (2007). Matrix metalloproteinase-mediated disruption of tight junction proteins in cerebral vessels is reversed by synthetic matrix metalloproteinase inhibitor in focal ischemia in rat. J. Cereb. Blood Flow Metab..

[20] Neuwelt E., Abbott N.J., Abrey L., Banks W.A., Blakley B., Davis T., Engelhardt B., Grammas P., Nedergaard M., Nutt J. (2008). Strategies to advance translational research into brain barriers. Lancet Neurol..

[21] Mun-Bryce S., Lukes A., Wallace J., Lukes-Marx M., Rosenberg G.A. (2002). Stromelysin-1 and gelatinase a are upregulated before TNF-α in LPS-stimulated neuroinflammation. Brain Res..

[22] Chung Y.C., Kim Y.S., Bok E., Yune T.Y., Maeng S., Jin B.K. (2013). MMP-3 contributes to nigrostriatal dopaminergic neuronal loss, BBB damage, and neuroinflammation in an MPTP mouse model of parkinson’s disease. Mediat. Inflamm..

[23] Ljubisavljevic S., Stojanovic I., Basic J., Vojinovic S., Stojanov D., Djordjevic G., Pavlovic D. (2015). The role of matrix metalloproteinase 3 and 9 in the pathogenesis of acute neuroinflammation. Implications for disease modifying therapy. J. Mol. Neurosci..

[24] Jalal F.Y., Yang Y., Thompson J., Lopez A.C., Rosenberg G.A. (2012). Myelin loss associated with neuroinflammation in hypertensive rats. Stroke.

[25] Brkic M., Balusu S., Libert C., Vandenbroucke R.E. (2015). Friends or foes: Matrix metalloproteinases and their multifaceted roles in neurodegenerative diseases. Mediat. Inflamm..

[26] Grossetete M., Phelps J., Arko L., Yonas H., Rosenberg G.A. (2009). Elevation of matrix metalloproteinases 3 and 9 in cerebrospinal fluid and blood in patients with severe traumatic brain injury. Neurosurgery.

[27] Kyriakis J.M. (1999). Activation of the AP-1 transcription factor by inflammatory cytokines of the TNF family. Gene Expr..

[28] Overall C.M., Lopez-Otin C. (2002). Strategies for MMP inhibition in cancer: Innovations for the post-trial era. Nat. Rev. Cancer.

[29] Rosenberg G.A. (2009). Matrix metalloproteinases and their multiple roles in neurodegenerative diseases. Lancet Neurol..

[30] Candelario-Jalil E., Taheri S., Yang Y., Sood R., Grossetete M., Estrada E.Y., Fiebich B.L., Rosenberg G.A. (2007). Cyclooxygenase inhibition limits blood-brain barrier disruption following intracerebral injection of tumor necrosis factor-α in the rat. J. Pharmacol. Exp. Ther..

[31] Kim E.M., Hwang O. (2011). Role of matrix metalloproteinase-3 in neurodegeneration. J. Neurochem..

[32] Woo M.S., Park J.S., Choi I.Y., Kim W.K., Kim H.S. (2008). Inhibition of MMP-3 or -9 suppresses lipopolysaccharide-induced expression of proinflammatory cytokines and inos in microglia. J. Neurochem..

[33] Frankowski J.C., DeMars K.M., Ahmad A.S., Hawkins K.E., Yang C., Leclerc J.L., Dore S., Candelario-Jalil E. (2015). Detrimental role of the EP1 prostanoid receptor in blood-brain barrier damage following experimental ischemic stroke. Sci. Rep..

[34] Eichler W., Friedrichs U., Thies A., Tratz C., Wiedemann P. (2002). Modulation of matrix metalloproteinase and TIMP-1 expression by cytokines in human RPE cells. Ophthalmol. Vis. Sci..

[35] Li H., Yoneda M., Takeyama M., Sugita I., Tsunekawa H., Yamada H., Watanabe D., Mukai T., Yamamura M., Iwaki M. (2010). Effect of infliximab on tumor necrosis factor-α-induced alterations in retinal microvascular endothelial cells and retinal pigment epithelial cells. J. Ocul. Pharmacol. Ther..

[36] Leu S.T., Batni S., Radeke M.J., Johnson L.V., Anderson D.H., Clegg D.O. (2002). Drusen are cold spots for proteolysis: Expression of matrix metalloproteinases and their tissue inhibitor proteins in age-related macular degeneration. Exp. Eye Res..

[37] Nemzek J.A., Hugunin K.M., Opp M.R. (2008). Modeling sepsis in the laboratory: Merging sound science with animal well-being. Comp. Med..

[38] Choi D.H., Kim J.H., Seo J.H., Lee J., Choi W.S., Kim Y.S. (2014). Matrix metalloproteinase-3 causes dopaminergic neuronal death through Nox1-regenerated oxidative stress. PLoS ONE.

[39] Christianson C.A., Fitzsimmons B.L., Shim J.H., Agrawal A., Cohen S.M., Hua X.Y., Yaksh T.L. (2012). Spinal matrix metalloproteinase 3 mediates inflammatory hyperalgesia via a tumor necrosis factor-dependent mechanism. Neuroscience.

[40] Pauly T., Ratliff M., Pietrowski E., Neugebauer R., Schlicksupp A., Kirsch J., Kuhse J. (2008). Activity-dependent shedding of the nmda receptor glycine binding site by matrix metalloproteinase 3: A putative mechanism of postsynaptic plasticity. PLoS ONE.

[41] Chu C.J., Gardner P.J., Copland D.A., Liyanage S.E., Gonzalez-Cordero A., Kleine Holthaus S.M., Luhmann U.F., Smith A.J., Ali R.R., Dick A.D. (2016). Multimodal analysis of ocular inflammation using the endotoxin-induced uveitis mouse model. Dis. Models Mech..

[42] Gadjanski I., Williams S.K., Hein K., Sattler M.B., Bahr M., Diem R. (2011). Correlation of optical coherence tomography with clinical and histopathological findings in experimental autoimmune uveoretinitis. Exp. Eye Res..

[43] Chu C.J., Herrmann P., Carvalho L.S., Liyanage S.E., Bainbridge J.W., Ali R.R., Dick A.D., Luhmann U.F. (2013). Assessment and in vivo scoring of murine experimental autoimmune uveoretinitis using optical coherence tomography. PLoS ONE.

[44] Fuentes M.E., Durham S.K., Swerdel M.R., Lewin A.C., Barton D.S., Megill J.R., Bravo R., Lira S.A. (1995). Controlled recruitment of monocytes and macrophages to specific organs through transgenic expression of monocyte chemoattractant protein-1. J. Immunol..

[45] Becker M.D., Garman K., Whitcup S.M., Planck S.R., Rosenbaum J.T. (2001). Inhibition of leukocyte sticking and infiltration, but not rolling, by antibodies to ICAM-1 and LFA-1 in murine endotoxin-induced uveitis. Ophthalmol. Vis. Sci..

[46] Choi D.H., Kim E.M., Son H.J., Joh T.H., Kim Y.S., Kim D., Flint Beal M., Hwang O. (2008). A novel intracellular role of matrix metalloproteinase-3 during apoptosis of dopaminergic cells. J. Neurochem..

[47] Gasche Y., Copin J.C., Sugawara T., Fujimura M., Chan P.H. (2001). Matrix metalloproteinase inhibition prevents oxidative stress-associated blood-brain barrier disruption after transient focal cerebral ischemia. J. Cereb. Blood Flow Metab..

[48] Zeni P., Doepker E., Schulze-Topphoff U., Huewel S., Tenenbaum T., Galla H.J. (2007). MMPs contribute to TNF-α-induced alteration of the blood-cerebrospinal fluid barrier in vitro. Am. J. Physiol. Cell Physiol..

[49] Sudharshan S., Ganesh S.K., Biswas J. (2010). Current approach in the diagnosis and management of posterior uveitis. Indian J. Ophthalmol..

[50] Kubota S., Kurihara T., Mochimaru H., Satofuka S., Noda K., Ozawa Y., Oike Y., Ishida S., Tsubota K. (2009). Prevention of ocular inflammation in endotoxin-induced uveitis with resveratrol by inhibiting oxidative damage and nuclear factor-kappab activation. Ophthalmol. Vis. Sci..

[51] Page-McCaw A., Ewald A.J., Werb Z. (2007). Matrix metalloproteinases and the regulation of tissue remodelling. Nat. Rev. Mol. Cell Biol..

[52] De Groef L., Andries L., Lemmens K., van Hove I., Moons L. (2015). Matrix metalloproteinases in the mouse retina: A comparative study of expression patterns and mmp antibodies. BMC Ophthalmol..

[53] Sternlicht M.D., Werb Z. (2001). How matrix metalloproteinases regulate cell behavior. Annu. Rev. Cell Dev. Biol..

[54] Rajagopalan S., Meng X.P., Ramasamy S., Harrison D.G., Galis Z.S. (1996). Reactive oxygen species produced by macrophage-derived foam cells regulate the activity of vascular matrix metalloproteinases in vitro. Implications for atherosclerotic plaque stability. J. Clin. Investig..

[55] Hsu H.Y., Wen M.H. (2002). Lipopolysaccharide-mediated reactive oxygen species and signal transduction in the regulation of interleukin-1 gene expression. J. Biol. Chem..

[56] Sasaki M., Ozawa Y., Kurihara T., Noda K., Imamura Y., Kobayashi S., Ishida S., Tsubota K. (2009). Neuroprotective effect of an antioxidant, lutein, during retinal inflammation. Ophthalmol. Vis. Sci..

[57] Kowluru R.A., Chan P.S. (2007). Oxidative stress and diabetic retinopathy. Exp. Diabetes Res..

[58] Neria F., del Carmen Serrano-Perez M., Velasco P., Urso K., Tranque P., Cano E. (2013). Nfatc3 promotes Ca^2+^ -dependent *Mmp3* expression in astroglial cells. Glia.

[59] Bouwmeester T., Bauch A., Ruffner H., Angrand P.O., Bergamini G., Croughton K., Cruciat C., Eberhard D., Gagneur J., Ghidelli S. (2004). A physical and functional map of the human TNF-α/NF-κB signal transduction pathway. Nat. Cell Biol..

[60] Skipor J., Szczepkowska A., Kowalewska M., Herman A.P., Lisiewski P. (2015). Profile of toll-like receptor mRNA expression in the choroid plexus in adult ewes. Acta Vet. Hung..

[61] Lu Y.C., Yeh W.C., Ohashi P.S. (2008). LPS/TLR4 signal transduction pathway. Cytokine.

[62] Jiang Q., Akashi S., Miyake K., Petty H.R. (2000). Lipopolysaccharide induces physical proximity between CD14 and toll-like receptor 4 (TLR4) prior to nuclear translocation of NF-κB. J. Immunol..

[63] Mallard C. (2012). Innate immune regulation by toll-like receptors in the brain. ISRN Neurol..

[64] Elner S.G., Petty H.R., Elner V.M., Yoshida A., Bian Z.M., Yang D., Kindezelskii A.L. (2005). TLR4 mediates human retinal pigment epithelial endotoxin binding and cytokine expression. Trans. Am. Ophthalmol. Soc..

[65] Alge-Priglinger C.S., Kreutzer T., Obholzer K., Wolf A., Mempel M., Kernt M., Kampik A., Priglinger S.G. (2009). Oxidative stress-mediated induction of MMP-1 and MMP-3 in human RPE cells. Ophthalmol. Vis. Sci..

[66] Chen H., Liu B., Lukas T.J., Neufeld A.H. (2008). The aged retinal pigment epithelium/choroid: A potential substratum for the pathogenesis of age-related macular degeneration. PLoS ONE.

[67] Tenenbaum T., Matalon D., Adam R., Seibt A., Wewer C., Schwerk C., Galla H.J., Schroten H. (2008). Dexamethasone prevents alteration of tight junction-associated proteins and barrier function in porcine choroid plexus epithelial cells after infection with streptococcus suis in vitro. Brain Res..

[68] Al-Saffar H., Lewis K., Liu E., Schober A., Corrigan J.J., Shibata K., Steiner A.A. (2013). Lipopolysaccharide-induced hypothermia and hypotension are associated with inflammatory signaling that is triggered outside the brain. Brain Behav. Immun..

[69] Leon L.R. (2004). Hypothermia in systemic inflammation: Role of cytokines. Front. Biosci..

[70] Kaplanski J., Nassar A., Sharon-Granit Y., Jabareen A., Kobal S.L., Azab A.N. (2014). Lithium attenuates lipopolysaccharide-induced hypothermia in rats. Eur. Rev. Med. Pharmacol. Sci..

[71] Vanlaere I., Libert C. (2009). Matrix metalloproteinases as drug targets in infections caused by gram-negative bacteria and in septic shock. Clin. Microbiol. Rev..

[72] Lipowsky H.H., Sah R., Lescanic A. (2011). Relative roles of doxycycline and cation chelation in endothelial glycan shedding and adhesion of leukocytes. Am. J. Physiol. Heart Circ. Physiol..

[73] Vandenbroucke R.E., Vanlaere I., van Hauwermeiren F., van Wonterghem E., Wilson C., Libert C. (2014). Pro-inflammatory effects of matrix metalloproteinase 7 in acute inflammation. Mucosal Immunol..

[74] Aerts J., Vandenbroucke R.E., Dera R., Balusu S., van Wonterghem E., Moons L., Libert C., Dehaen W., Arckens L. (2015). Synthesis and validation of a hydroxypyrone-based, potent, and specific matrix metalloproteinase-12 inhibitor with anti-inflammatory activity in vitro and in vivo. Mediat. Inflamm..

[75] Vandenbroucke R.E., Dejonckheere E., van Hauwermeiren F., Lodens S., de Rycke R., van Wonterghem E., Staes A., Gevaert K., Lopez-Otin C., Libert C. (2013). Matrix metalloproteinase 13 modulates intestinal epithelial barrier integrity in inflammatory diseases by activating TNF. EMBO Mol. Med..

[76] Shen D.F., Buggage R.R., Eng H.C., Chan C.C. (2000). Cytokine gene expression in different strains of mice with endotoxin-induced uveitis (EIU). Ocul. Immunol. Inflamm..

[77] Planck S.R., Huang X.N., Robertson J.E., Rosenbaum J.T. (1994). Cytokine mRNA levels in rat ocular tissues after systemic endotoxin treatment. Ophthalmol. Vis. Sci..

[78] Hoekzema R., Murray P.I., van Haren M.A., Helle M., Kijlstra A. (1991). Analysis of interleukin-6 in endotoxin-induced uveitis. Ophthalmol. Vis. Sci..

[79] Leung K.W., Barnstable C.J., Tombran-Tink J. (2009). Bacterial endotoxin activates retinal pigment epithelial cells and induces their degeneration through IL-6 and IL-8 autocrine signaling. Mol. Immunol..

[80] De Vos A.F., Klaren V.N., Kijlstra A. (1994). Expression of multiple cytokines and IL-1RA in the uvea and retina during endotoxin-induced uveitis in the rat. Ophthalmol. Vis. Sci..

[81] Ley K., Laudanna C., Cybulsky M.I., Nourshargh S. (2007). Getting to the site of inflammation: The leukocyte adhesion cascade updated. Nat. Rev. Immunol..

[82] Li X., Gu X., Boyce T.M., Zheng M., Reagan A.M., Qi H., Mandal N., Cohen A.W., Callegan M.C., Carr D.J. (2014). Caveolin-1 increases proinflammatory chemoattractants and blood-retinal barrier breakdown but decreases leukocyte recruitment in inflammation. Ophthalmol. Vis. Sci..

[83] Benhar I., Reemst K., Kalchenko V., Schwartz M. (2016). The retinal pigment epithelium as a gateway for monocyte trafficking into the eye. EMBO J..

[84] Goureau O., Bellot J., Thillaye B., Courtois Y., de Kozak Y. (1995). Increased nitric oxide production in endotoxin-induced uveitis. Reduction of uveitis by an inhibitor of nitric oxide synthase. J. Immunol..

[85] Shi F., Duan S., Cui J., Yan X., Li H., Wang Y., Chen F., Zhang L., Liu J., Xie X. (2014). Induction of matrix metalloproteinase-3 (MMP-3) expression in the microglia by lipopolysaccharide (LPS) via upregulation of glycoprotein nonmetastatic melanoma B (GPNMB) expression. J. Mol. Neurosci..

[86] Sinfield J.K., Das A., O’Regan D.J., Ball S.G., Porter K.E., Turner N.A. (2013). P38 MAPK alpha mediates cytokine-induced IL-6 and MMP-3 expression in human cardiac fibroblasts. Biochem. Biophys. Res. Commun..

[87] Neuder L.E., Keener J.M., Eckert R.E., Trujillo J.C., Jones S.L. (2009). Role of p38 MAPK in LPS induced pro-inflammatory cytokine and chemokine gene expression in equine leukocytes. Vet. Immunol. Immunopathol..

[88] Guha M., O’Connell M.A., Pawlinski R., Hollis A., McGovern P., Yan S.F., Stern D., Mackman N. (2001). Lipopolysaccharide activation of the MEK-ERK1/2 pathway in human monocytic cells mediates tissue factor and tumor necrosis factor alpha expression by inducing Elk-1 phosphorylation and EGR-1 expression. Blood.

[89] Steenport M., Khan K.M., Du B., Barnhard S.E., Dannenberg A.J., Falcone D.J. (2009). Matrix metalloproteinase (MMP)-1 and MMP-3 induce macrophage MMP-9: Evidence for the role of TNF-α and cyclooxygenase-2. J. Immunol..

[90] Gearing A.J., Beckett P., Christodoulou M., Churchill M., Clements J.M., Crimmin M., Davidson A.H., Drummond A.H., Galloway W.A., Gilbert R. (1995). Matrix metalloproteinases and processing of pro-TNF-α. J. Leukoc. Biol..

[91] Konari K., Sawada N., Zhong Y., Isomura H., Nakagawa T., Mori M. (1995). Development of the blood-retinal barrier in vitro: Formation of tight junctions as revealed by occludin and ZO-1 correlates with the barrier function of chick retinal pigment epithelial cells. Exp. Eye Res..

[92] Russ P.K., Davidson M.K., Hoffman L.H., Haselton F.R. (1998). Partial characterization of the human retinal endothelial cell tight and adherens junction complexes. Ophthalmol. Vis. Sci..

[93] Poulaki V., Iliaki E., Mitsiades N., Mitsiades C.S., Paulus Y.N., Bula D.V., Gragoudas E.S., Miller J.W. (2007). Inhibition of Hsp90 attenuates inflammation in endotoxin-induced uveitis. FASEB J..

[94] Zech J.C., Pouvreau I., Cotinet A., Goureau O., Le Varlet B., de Kozak Y. (1998). Effect of cytokines and nitric oxide on tight junctions in cultured rat retinal pigment epithelium. Ophthalmol. Vis. Sci..

[95] Vanden Berghe T., Hulpiau P., Martens L., Vandenbroucke R.E., van Wonterghem E., Perry S.W., Bruggeman I., Divert T., Choi S.M., Vuylsteke M. (2015). Passenger mutations confound interpretation of all genetically modified congenic mice. Immunity.

[96] Verslegers M., van Hove I., Dekeyster E., Gantois I., Hu T.T., D’Hooge R., Arckens L., Moons L. (2015). MMP-2 mediates purkinje cell morphogenesis and spine development in the mouse cerebellum. Brain Struct. Funct..

[97] Van Hove I., Verslegers M., Buyens T., Delorme N., Lemmens K., Stroobants S., Gantois I., D’Hooge R., Moons L. (2012). An aberrant cerebellar development in mice lacking matrix metalloproteinase-3. Mol. Neurobiol..

[98] Saito M., Barbazetto I.A., Spaide R.F. (2013). Intravitreal cellular infiltrate imaged as punctate spots by spectral-domain optical coherence tomography in eyes with posterior segment inflammatory disease. Retina.

[99] Prusky G.T., Alam N.M., Beekman S., Douglas R.M. (2004). Rapid quantification of adult and developing mouse spatial vision using a virtual optomotor system. Investig. Ophthalmol. Vis. Sci..

[100] Douglas R.M., Alam N.M., Silver B.D., McGill T.J., Tschetter W.W., Prusky G.T. (2005). Independent visual threshold measurements in the two eyes of freely moving rats and mice using a virtual-reality optokinetic system. Vis. Neurosci..

[101] De Groef L., Dekeyster E., Geeraerts E., Lefevere E., Stalmans I., Salinas-Navarro M., Moons L. (2016). Differential visual system organization and susceptibility to experimental models of optic neuropathies in three commonly used mouse strains. Exp. Eye Res..

